# Meta-Analytic Evidence for a Reversal Learning Effect on the Iowa Gambling Task in Older Adults

**DOI:** 10.3389/fpsyg.2017.01785

**Published:** 2017-10-11

**Authors:** Rita Pasion, Ana R. Gonçalves, Carina Fernandes, Fernando Ferreira-Santos, Fernando Barbosa, João Marques-Teixeira

**Affiliations:** ^1^Laboratory of Neuropsychophysiology, Faculty of Psychology and Educational Sciences, University of Porto, Porto, Portugal; ^2^Católica Porto Business School, Universidade Católica Portuguesa, Porto, Portugal; ^3^Faculty of Medicine, University of Porto, Porto, Portugal

**Keywords:** Iowa Gambling Task, decision-making, risk, uncertainty, aging, older adults, neuropsychology

## Abstract

Iowa Gambling Task (IGT) is one of the most widely used tools to assess economic decision-making. However, the research tradition on aging and the Iowa Gambling Task (IGT) has been mainly focused on the overall performance of older adults in relation to younger or clinical groups, remaining unclear whether older adults are capable of learning along the task. We conducted a meta-analysis to examine older adults' decision-making on the IGT, to test the effects of aging on reversal learning (45 studies) and to provide normative data on total and block net scores (55 studies). From the accumulated empirical evidence, we found an average total net score of 7.55 (±25.9). We also observed a significant reversal learning effect along the blocks of the IGT, indicating that older adults inhibit the prepotent response toward immediately attractive options associated with high losses, in favor of initially less attractive options associated with long-run profit. During block 1, decisions of older adults led to a negative gambling net score, reflecting the expected initial pattern of risk-taking. However, the shift toward more safe options occurred between block 2 (small-to-medium effect size) and blocks 3, 4, 5 (medium-to-large effect size). These main findings highlight that older adults are able to move from the initial uncertainty, when the possible outcomes are unknown, to decisions based on risk, when the outcomes are learned and may be used to guide future adaptive decision-making.

## Introduction

Decision-making is a fundamental process in everyday life and subject to major changes over the lifespan. According to a recent meta-analysis, early adolescents show a pattern of risk-seeking behavior compared to mid-late adolescents, despite similar performances in decision-making between children and adolescents (Defoe et al., 2015). Moreover, adolescents were found to be more risk-seeking in tasks with immediate feedback compared to adults (Defoe et al., 2015).

A meta-analysis in healthy older adults was further conducted by Mata et al. (2011), while differentiating participants' decision-making performance in contexts of uncertainty and risk. Uncertainty refers to circumstances in which the probabilities of the possible outcomes are unknown, while in decisions under risk the outcomes and probabilities are given in advance (Ellsberg, 1961; Kahneman and Tversky, 1979; Mata et al., 2011). Older adults seem to engage more often in disadvantageous decisions than younger adults, but only under uncertainty (Mata et al., 2011). Under risk, younger and older adults showed similar patterns of decision-making (Mata et al., 2011). Interestingly, older adults engage less in risky activities compared to younger adults, and are more responsive to warnings about potential risks (Rolison et al., 2016). The aging-related reduction in risk-taking seems to occur steeply for financial and recreational decisions, but smoothly for ethical and health-related decisions (Rolison et al., 2013). It seems that, under risk, older adults respond to threat levels with increased cautiousness (Rolison et al., 2013, 2016), but the threat level may be difficult to identify without previous information, as occurs in decision-making under uncertainty (Mata et al., 2011).

The processes and mechanisms that may explain the abovementioned age differences in decisions under risk and uncertainty are still poorly understood. The Iowa Gambling Task (IGT) (Bechara et al., 1994) may advance our knowledge on the differential patterns of performance under risk and uncertainty, since the decisions along this task are expected to move from uncertainty to risk (Brand et al., 2007).

In the IGT, participants are asked to choose a card from four different decks to win as much money as possible. While performing the task it is expected that participants learn to discriminate advantageous (Decks C and D) from disadvantageous decks (Decks A and B). However, learning during the IGT requires more than just adjusting behavior as a function of reliable feedback signaling long-term correct and incorrect responses. Considering that high rewards in the IGT are included in the disadvantageous decks, the prepotent response is initially oriented to the decks that are also associated with increased losses (Kovalchik and Allman, 2006). Adaptive behavior requires the inhibition of the prepotent response, as participants learn to forego the high monetary rewards (immediately attractive options that are also associated with high losses) in favor of the low to moderate monetary rewards (initially less attractive options that are associated with reduced losses and long-run profit). The shift in the prepotent response during the learning process is conceptualized as the reversal learning effect (Kovalchik and Allman, 2006). The difference between the number of disadvantageous (Decks A and B) and advantageous choices (Decks C and D) is considered a Gambling Index—the total net score (Bechara et al., 1994)—that captures the reversal learning effect and the adaptive course of action. Since the implicit feedback in the first half of the task is considered a close correlate of the uncertainty experienced in real-life (Bechara et al., 1994; Verdejo-Garcia et al., 2006; Bechara, 2007; Brevers et al., 2013), the reversal learning effect may unveil the moment in which participants learn the advantageous strategy. Brand et al. (2007) reported that only the last trials of IGT were correlated with the performance under risk, supporting that decks probabilities are learned along the task.

The starting point to conceptualize the shift from uncertainty to risk is grounded on Damásio's Somatic Marker Hypothesis (Damasio, 1994). Damasio (1994) proposed that the affective signals generated from the match between choices and associated outcomes guide subsequent decisions, by biasing the decision to the options associated with positive affective states. The affective states are detected by the limbic system, particularly, the amygdala. During the first trials, the limbic areas trigger the affective values of gains and losses, and generate the automatic somatic states (primary inducers) (Bechara et al., 2003; Bechara and Damasio, 2005). Interestingly, levels of uncertainty are positively associated with amygdala activation, suggesting that this structure is recruited to detect relevant information when the probabilities are unknown (Hsu et al., 2005).

An affective executive system—the hot executive functioning (EF)—also accounts to detect monetary rewards and losses under uncertainty. The hot EF is defined as the set of abilities that regulate emotional awareness, impulsive reactions and goal achievement, by integrating emotional, affective, and visceral processes (Miyake et al., 2000; Zelazo and Müller, 2002; Séguin et al., 2007; Brevers et al., 2013). The incentive saliency measured under uncertainty is monitored by the hot executive functioning to signal the best outcomes. Then, the information from primary inducers is accommodated in the memory systems (Bechara et al., 2003; Bechara and Damasio, 2005), and the hot EF assists the cold EF in an integrated decision process (Zelazo and Müller, 2002; Séguin et al., 2007; Brevers et al., 2013). The cold EF is conceptualized as the cognitive determinant of risk and gains, updating and maintaining the information in working memory (Zelazo and Müller, 2002; Séguin et al., 2007; Brevers et al., 2013). The hot and cold EF interplay is a critical process to plan the necessary changes to future choices (Zelazo and Müller, 2002; Brevers et al., 2013). The ventromedial prefrontal cortex (vmPFC) will be critical to guide future adaptive decision-making, mediating the secondary inducers—somatic states generated by the recall of emotional events (Bechara et al., 2000, 2003; Bechara and Damasio, 2005). The vmPFC activation is associated with global IGT performance (Northoff et al., 2006; Lawrence et al., 2009; Li et al., 2010) and, interestingly, with performance in the final trials (Northoff et al., 2006), suggesting that the vmPFC becomes less dependent on amygdala-driven autonomic responses at the end of the task (Bechara et al., 2003).

Functional age-related changes in brain areas implicated in decision-making [e.g., insula and anterior cingulate cortex (Good et al., 2001), superior temporal sulcus (Sowell et al., 2003), dorsal and ventral striatum (Raz et al., 2005; Walhovd et al., 2005), prefrontal (West, 2000), and orbitofrontal cortex (Resnick et al., 2007)] suggest that older adults exhibit less resources to decide adaptively. EF, mainly dependent on prefrontal areas, are particularly vulnerable to age-related cognitive decline (Best et al., 2009). From the 7th decade of life, a detrimental effect is found in several executive domains, such as response inhibition, planning, and set shifting (Best et al., 2009), that are important functions to reversal learning. Older adults tend to make more perseverative errors, which indicate an inability to plan future behavior in function of previous feedback and a failure to inhibit an activated response pattern that as proven to be disadvantageous.

A reversal learning effect in older players performing the IGT is not detected when compared to younger players (Kovalchik and Allman, 2006). Kovalchik and Allman (2006) proposed that the lack of an initial preference in older adults compromises the subsequent process of reversal learning. Decision-making and reversal learning seem, therefore, to become inoperative with age, suggesting that a random selection strategy may be guiding decision-making in elderly (Kovalchik and Allman, 2006).

Steingroever et al. (2013) also proposed that IGT performance on healthy groups are characterized by slow learning processes, and 100 trials are not sufficient to learn to discriminate the safe over the risky options. The infrequent occurrence of losses in decks B and D provides little information to learn that deck B should be avoided. Moreover, with the exception of the deck A, the remaining decks seem to have too similar outcomes (Steingroever et al., 2013). Participants fail, therefore, to distinguish bad from good decks, failing to progress from an initial stage of exploration to a later stage of exploitation. The limitations of the learning processes expected to occur during the IGT may be particularly observed in older groups, since this group have increased difficulty in discriminating negative from positive outcomes in reinforcement learning tasks. The reduced Feedback-Related Negativity (FRN) amplitude was found to be similar after losses and gains, suggesting a decreased focus of the monitoring system in classifying the outcomes according to task-specific goals (Hämmerer et al., 2011). In fact, older adults need more trials to identify the option more likely to be rewarded, particularly when differences in reward likelihood between choices are small (Hämmerer et al., 2011).

The revised literature documents that older adults show detrimental changes in decision-making brain-related areas that may compromise critical functions as EF, reversal and reinforcement learning that are critical processes to decision-making under uncertainty. However, and considering that decisions under risk are similar (Mata et al., 2011) or even improved compared to younger adults (Rolison et al., 2013, 2016), it remains unclear whether older adults are capable of learning adaptive strategies and move from uncertainty toward risk, that is, to integrate affective automatic responses in memory and rational analytical systems that facilitate future adaptive decision-making.

The current meta-analysis aims to address this gap in the literature. The IGT includes both initial stages of exploration (decisions under uncertainty) and later stages of exploitation (decisions under risk) (Brand et al., 2007; Steingroever et al., 2013), providing a comprehensive analysis of decision-making. This allows extending Mata et al.'s (2011) results, which were obtained with tasks assessing decision-making under risk and under uncertainty independently. Also, Mata et al.'s (2011) conclusions are retrieved from studies with a between-group design (older vs. younger groups), from which we cannot infer directly that older adults are not capable of learning.

For this purpose, we have meta-analyzed the performance of older adults along the IGT blocks. The within-subject design of our meta-analysis allow monitoring the participants' performance along the task and to isolate the reversal learning effect. The analysis of the shift from uncertainty to risk is of great importance, since older adults' difficulties in decision-making appear to be restricted to uncertainty (Mata et al., 2011). We hypothesize that the contrasting pattern of performance under risk and uncertainty is explained by the lack of a reversal learning effect in older adults (Kovalchik and Allman, 2006). The reversal learning effect is required to perform adaptively in tasks under uncertainty, and subsequently to move to a context of decision-making under risk, in which the task contingencies have been learned and may guide future adaptive decisions.

This hypothesis constitutes an innovative approach to the IGT, since the results are typically analyzed in terms of the total net score, disregarding the dynamics of learning that occurs within the task. Finally, we also provide normative data on older adults' performance from the literature reviewed, namely a group reference criterion to compare individual values of IGT total and block net scores.

## Methods

The current meta-analysis followed the PRISMA Statement guidelines for reporting systematic reviews and meta-analyses (Moher et al., 2009).

### Eligibility criteria

The focus of the systematic search was studies that assessed economic decision-making processes in older adults with the IGT.

As inclusion criteria, the studies had to: (1) describe empirical results; (2) report the Bechara et al.'s (1994) original version of the IGT, in its manual or computerized versions; (3) include a sample of healthy older adults (mean age ≥ to 55 years old and standard deviation < to 10). Mean age criteria was based on Denburg's et al. (2005) cut off, and standard deviation criteria was defined to avoid samples with a large interval of age.

Studies were excluded if: (4) none of the parameters of the current review (total and block IGT net scores) were reported; and (5) contained overlapping results.

To avoid publication bias, we considered unpublished results, but none were retained after the application of inclusion/exclusion criteria.

### Study selection

PubMed, EBSCOhost (Academic Search Complete, PsycARTICLES, Psychology and Behavioral Sciences Collection), and Web of Knowledge databases were used to identify papers published since the first administration of IGT (Bechara et al., 1994) (1994–September 2016).

The search expression, limited to titles and abstracts in English, was (neurodegenerative OR Alzheimer OR Parkinson OR Huntington OR dementia OR “mild cognitive impairment” OR “frontotemporal dementia” OR ageing OR aging OR “older adults” OR elderly) AND (“Iowa Gambling Task”). Neurodegenerative disorders were included in the search expression to identify papers using healthy adults as controls.

The selection of the studies included the following steps: (1) combination of search results from different databases and removal of duplicates; (2) assessment of inclusion criteria by two independent raters (RP, CF), considering the abstract and full text. Disagreements were resolved by consensus; (3) reference lists were screened to identify additional relevant papers; (4) authors were contacted to provide missing information; (5) papers with missing or repeated data were excluded.

### Data collection and variables extracted

During the assessment of inclusion criteria, the inter-rater agreement Cohen's kappa was used to compare the agreement between the researchers, revealing an almost perfect agreement (*K* = 0.95).

A standardized coding form was then developed to systematically collect the main parameters of analysis. This process was conducted by two independent researchers (RP and ARG).

The extracted variables from the included studies were: final sample size (*n*), gender (*n* females), age (mean and standard deviation), years of education (mean and standard deviation), task administration (computerized or manual), compensation (none, fixed, proportional to the performance), total and blocks net score (means and standard deviations).

### Data analysis

The quantitative results obtained from total and block net scores were used to achieve our main goals.

To compute the normative data, the standard errors of the mean extracted from the figures were first converted to standard deviation by multiplying the standard error of the mean by the square root of the sample size (Higgins and Green, 2011). Pooled means (*M*_pooled_) and pooled standard deviations (*SD*_pooled_) were then calculated for each study. These pooled parameters compose a single unit of analysis in which larger sample sizes are proportionally represented by a greater effect on the overall estimate, which improves the estimate precision and allows to compare independent sample estimates.

To explore the effects of aging on reversal learning, we computed the magnitude of the effect size from the difference between block net scores, always in reference to the baseline (block 1; B1). All analyses were conducted on Comprehensive Meta-Analysis software (3.0; Biostat, U.S.A).

The meta-analytic methods were performed in accordance to a within-subject design, as the data for the same participants was entered for more than one condition, introducing statistical dependence between the conditions. The work on statistical methods for meta-analysis has been more focused on independent sample sizes, whereas repeated measures received more limited attention. The use of the same methods to calculate independent and dependent effects is not recommended, as it introduces significant estimate bias on within-subject designs (Dunlap et al., 1996; Morris and DeShon, 2002). The calculation of the within-subject effect size is dependent on the value of the correlation between conditions, in addition to the means and standard deviations of each condition. However, this value is rarely provided in research reports. Indeed, none of the reviewed studies reported this correlation, because the main interest was to test group differences between the older group and the younger or clinical groups. This issue has a critical relevance to the current meta-analysis, as the magnitude of the effect size depends on whether the correlations between the conditions are smaller, larger, or equal to 0.5 (Morris and DeShon, 2002; Ferreira-Santos, in press).

Given the issues outlined above, we opted to impute the estimated correlation from the databases provided by the authors, where the correlation values between all blocks were available (seven samples). Correlations were pooled by using the weighted average of Z-transformed coefficient coefficients, which was then transformed back into a correlation coefficient via Fisher's Z inverse transformation (Silver and Dunlap, 1987). Because the distribution of *Z* is approximately normal, this method tends to be less biased than a simple arithmetic average, which distribution becomes negatively skewed as the correlation is larger than zero, particularly when including small samples (Silver and Dunlap, 1987).

All the seven samples used to impute the correlation coefficient revealed a low correlation between blocks: *r* = −0.107 to 0.219 (B2-B1); *r* = −0.039 to 0.379 (B3-B1); *r* = −0.155 to 0.198 (B4-B1); *r* = −0.024 to 0.277 (B5-B1). This resulted in an imputed *r* value of 0.007 in B2-B1, of 0.086 in B3-B1, of 0.018 in B4-B1, and of 0.068 in B5-B1. For the seven samples where the *r* value was available, the original value was maintained in accordance to the performances between blocks. To assess whether the variation in the correlation value would modify the reported effect size, a sensitivity analysis using a range of plausible correlations was conducted using moderated (0.50) and high (0.80) correlations.

From the imputed correlation coefficients, we calculated the Hedge's *g*. This method prevents the overestimation of the absolute value of the effect size parameter in studies with small samples (Hedges, 1981), as frequently observed for Cohen's *d* (Cohen, 1988).

High scores on *g* indicate a positive net outcome (i.e., better decisions on later decks when compared to the first deck), while negative values are associated with negative outcomes and disadvantageous behavior.

### Heterogeneity analysis

The heterogeneity analysis allows testing the consistency of results across included studies. Statistical heterogeneity between studies is considered inevitable, since methodological diversity always occurs (Higgins and Green, 2011).

The variability between studies, that is, differences in effect sizes that are caused by other factors than chance (sampling error), was tested using the *Q* test (Cochran, 1954) and *I*^2^ (Higgins and Green, 2011). The significance of *Q* indicates the presence of heterogeneity, while the *I*^2^ describes the percentage of the variability in effect estimates that is due to heterogeneity. Heterogeneity was present in the current meta-analysis, suggesting that there is in fact more than one true effect sizes at the population-level. Considering this, we may not assume that individual effect sizes are measures of a single population effect size (fixed-effects models). The alternative is to incorporate the heterogeneity in the random-effect models, where individual estimates are measures of a distribution of possible population-level effect sizes (Field, 2001; Schmidt et al., 2009; Higgins and Green, 2011). Providing a hyperparameter of the population distribution, random-effect models allow us to generalize the findings to the population, whereas inferences based on fixed-effects models are restricted to the set of the studies reviewed (Schmidt et al., 2009).

### Moderation analysis

To further explore the factors that may be accounting to the heterogeneity in results, we performed a moderation analysis.

Sample (proportion of females, age and years of education) and task variables (administration and compensation) are variables systematically identified in the literature and thought to modulate the performance in the IGT (for a review see Fernie and Tunney, 2006; Areias et al., 2013). However, the lack of variability in task administration and compensation variables, in addition to restricted information from several variables of interest, conditioned the assessment of moderation effects of these variables. Consequently, these variables were only used to better characterize the studies in which the use of the normative data may be particularly relevant.

Regarding the sample characteristics, age, years of education, and percentage of females were considered as continuous moderator variables. Independent meta-regressions across block performance were conducted to test the learning effect, when moderated by age, years of education, and the percentage of females in the sample.

## Results

### Study inclusion

**Figure 1:**
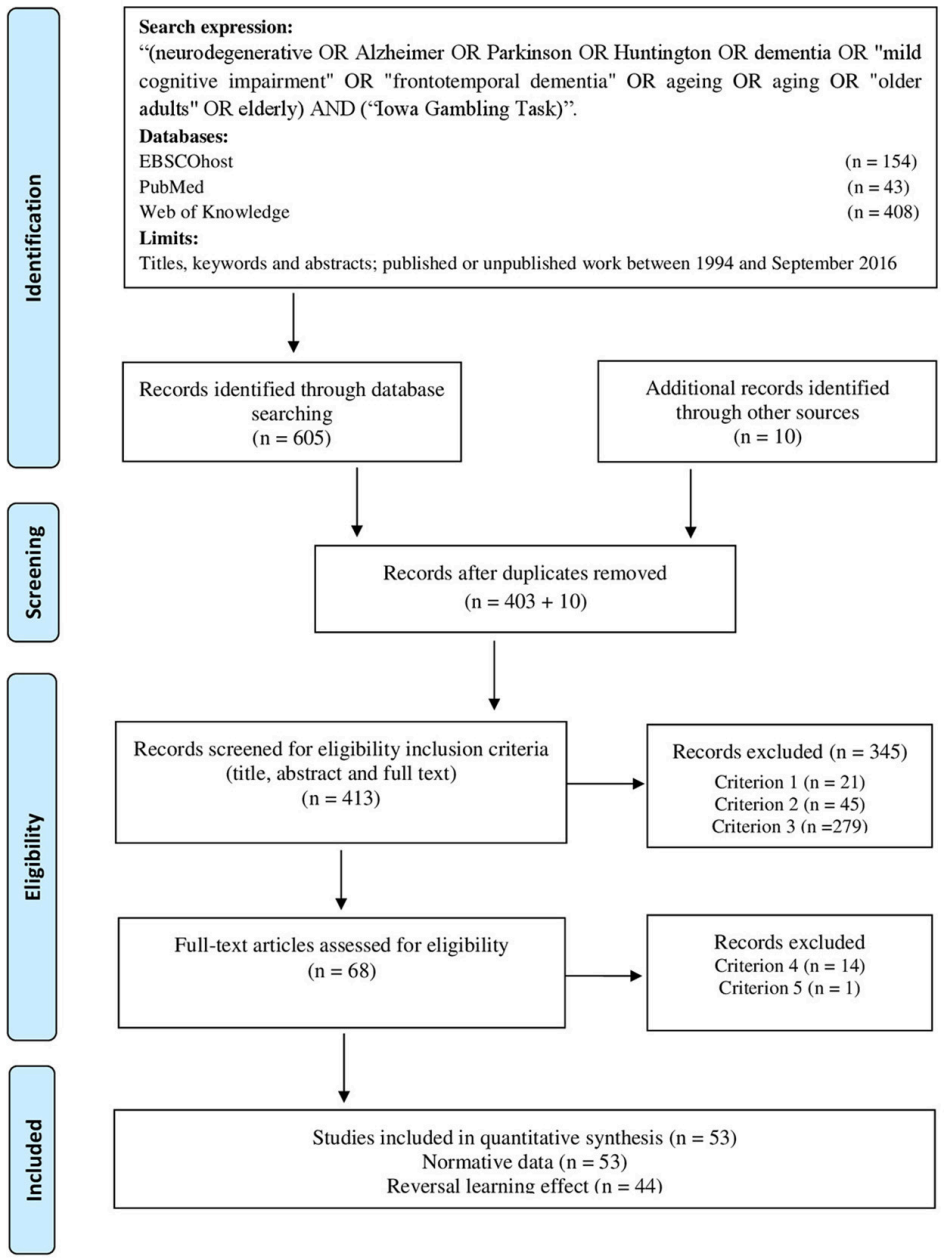
PRISMA flow diagram.

Detailed information on the study selection process is described in the PRISMA Flow Diagram (Figure 1).

A total of 403 non-duplicated articles were found and 10 studies were added by cross-reference check.

In nine studies, it was not possible to assess inclusion criteria. Authors were then contacted and asked to provide more detailed information about the mean age and respective standard deviation of the samples. Responses were not obtained for one study (response rate = 88.9%) that was, therefore, excluded based on inclusion criterion 3.

Twenty-one studies did not meet the inclusion criterion 1, 45 the inclusion criterion 2, and 279 studies the inclusion criterion 3.

For the 68 eligible papers, only three studies reported all the required information to test our main hypothesis. For the remaining studies, the authors were contacted. Data was no longer available for five studies, but additional information was provided for 25 studies. Of note, Caselli et al. (2011) kindly sent to us a larger dataset of the published study. Lamar also provided us with a more recent database from Visagan et al.'s (2012) study. This latter paper only reports IGT performance for a younger group, but the authors kindly authorized us to report the performance of the healthy older adults collected at the time. For the studies with no response or with no information available, the total and block net scores were extracted from the graphical illustrations using Engauge Digitizer software (V9.8, https://markummitchell.github.io/engauge-digitizer/). However, 14 articles did not contain the required information and were removed from the analysis (exclusion criterion 4).

The contact with the authors and the overlap of the outcome measures also allowed us to identify repeated data across studies (exclusion criterion 5). One study was removed.

Fifty-three articles (55 cells) were retained to calculate the normative data for the IGT performance in aging. A subset of 44 studies (45 cells) was used to test the effect of aging on the reversal learning effect. All the studies were published between 2002 and 2016^1^.

### Sample

The data from 1977 older adults (55% female) were used to calculate normative data on the IGT performance (Table 1). The mean age of the sample was 68.2 years and the mean years of formal education was 13.2.

**Table 1:** Study characteristics and normative total and block net scores for the 55–79 age range.

	**Sample**				**Task**		**Iowa Gambling Task net score**												
	**Size**		**Age**	**Education**	**Computorized**	**Compensation**	**Block 1**		**Block 2**		**Block 3**		**Block 4**		**Block 5**		**Total net score**		
	***n***	***Female***	***M***	***M***			***M* (*k* = 51)**	***SD* (*k* = 45)**	***M* (*k* = 51)**	***M* (*k* = 45)**	***SD* (*k* = 31)**	***SD* (*k* = 45)**	***M* (*k* = 51)**	***SD* (*k* = 45)**	***M* (*k* = 51)**	***SD* (*k* = 45)**	***M* (*k* = 43)**	***SD* (*k* = 31)**	**Net outcome**
MacPherson et al., 2002	30	15	69.9	12.4		–	−4.11	8.23	–0.76	10.1	−0.18	5.19	−1.01	10.18	0.39	8.74	–	–	–
Rosi et al., 2016	72	43	68.0	14.6	Yes	–	−1.92	8.99	3.78	7.98	3.78	8.22	4.75	8.99	4.00	10.4	14.39	28.9	↑
Auzou et al., 2014	18	7	68.0	1.40		–	−1.56	6.38	−1.56	5.14	−3.00	7.61	0.00	7.27	0.22	10.3	−5.24	24.9	↓
Ibarretxe-Bilbao et al., 2009	24	8	57.8	13.0	Yes	–	−1.17	3.80	1.04	4.29	9.15	5.62	12.1	6.45	14.9	6.54	36.5	22.7	↑
Mapelli et al., 2014	15	4	60.7	11.4	Yes	–	−5.27	10.3	0.03	10.2	2.98	10.1	7.20	10.27	9.89	10.9	–	–	–
Poletti et al., 2010	25	11	65.4	9.30	Yes	–	−2.33	2.61	−0.27	3.01	1.33	3.26	3.20	1.97	4.20	4.54	6.10	6.8	↑
Torralva et al., 2009	14	7	65.5	13.9	Yes	–	1.56	7.48	0.91	6.38	5.67	6.36	5.32	6.38	8.89	8.59	–	–	–
Icellioglu, 2015	30	15	66.3	9.54	Yes	No	−4.48	6.04	−0.72	5.21	3.87	3.23	1.57	5.44	1.84	6.79	3.45	12.8	↑
Schiebener and Brand, 2016	42	26	67.5	–		–	−2.50	5.41	1.45	6.65	1.14	8.17	2.10	8.79	3.74	9.50	−0.19	25.1	↓
Carvalho et al., 2012	40	30	67.4	14.7	Yes	–	−0.15	5.44	0.95	5.54	2.45	6.26	–0.15	6.67	0.55	8.37	3.93	21.6	↑
Euteneuer et al., 2009	23	11	64.4	11.7	Yes	–	−3.65	3.70	1.48	5.53	3.00	7.15	2.17	6.95	2.52	7.94	5.48	22.7	↑
Kobayakawa et al., 2008	22	9	67.6	14.4	Yes	–	1.09	3.58	0.00	4.94	0.64	5.10	3.64	4.392	1.73	6.60	4.90	2.60	↑
Bakos et al., 2008	10	9	62.0	14.1	Yes	–	0.01	–	1.23	–	2.69	–	0.84	–	4.92	–	15.4	23.1	↑
Czernecki et al., 2002	28	10	58.1	12.7	Yes	–	−4.79	3.71	0.14	6.53	0.79	8.06	5.00	9.97	6.43	10.9	7.60	4.20	↑
Balconi et al., 2014a	42	24	57.0	13.6	Yes	–	0.76	14.2	1.82	14.6	7.75	14.8	8.98	14.1	12.5	13.8	–	–	–
Caselli et al., 2011	110	77	63.4	16.4		–	−1.39	9.77	4.45	8.89	5.40	9.74	5.98	8.99	4.15	10.6	18.6	26.8	↑
Evens et al., 2015	32	9	65.3	10.7	Yes	–	−0.09	7.33	−1.44	6.61	−2.31	5.74	−1.22	6.08	−2.91	6.74	−7.31	20.8	↓
Kloeters et al., 2013	28	12	64.2	13.7	Yes	–	−1.10	8.40	2.80	7.10	5.90	9.50	7.30	9.30	4.00	8.70	18.9	28.9	↑
Smart and Krawitz, 2015	25	10	69.9	16.9	Yes	–	−0.34	0.76	2.81	7.68	7.50	16.9	6.98	15.1	3.98	9.08	–	–	–
Bertoux et al., 2013	30	15	67.2	10.7	Yes	–	−2.40	3.90	−0.13	5.70	0.80	5.80	−0.20	8.00	−1.33	7.70	−3.2	22.9	↓
Isella et al., 2008	40	22	65.4	8.70		–	−0.96	24.0	1.03	39.4	2.72	52.5	3.59	59.2	0.81	38.0	7.1	19.6	↑
Zamarian et al., 2008	52	34	69.3	–	Yes	–	−4.00	5.57	2.15	6.16	5.27	7.79	5.54	8.76	7.35	8.28	16.4	20.7	↑
Delazer et al., 2012	45	23	63.9	11.8	Yes	–	−3.44	11.5	0.56	19.2	4.79	8.26	7.90	9.74	8.77	8.84	18.4	27.54	↑
Balconi et al., 2014b	39	17	56.4	13.9	Yes	–	−0.11	0.44	1.01	0.62	3.60	0.87	5.90	0.75	8.02	0.69	–	–	–
Gleichgerrcht et al., 2012	14	7	65.5	13.9		–	1.50	7.60	0.80	6.40	5.60	6.60	5.20	6.70	8.80	8.50	21.9	19.9	↑
Torralva et al., 2007	10	6	63.5	13.5	Yes	–	1.31	8.38	0.87	6.86	5.69	7.30	5.64	7.51	8.98	9.56	22.9	–	↑
Baena et al., 2010	39	–	69.9	–	Yes	–	–0.26	13.4	2.31	14.4	3.13	16.8	1.44	17.8	2.10	16.9	1.74	15.9	↑
Sasai et al., 2012	34	13	64.0	13.0	Yes	–	–0.48	3.28	0.51	1.91	1.99	1.77	1.36	1.37	3.55	2.87	6.90	16.6	↑
Ottaviani and Vandone, 2011	114	91	59.1	–	Yes	–	–3.84	6.09	−1.95	6.98	−1.47	8.95	−1.95	8.92	−0.23	9.49	−9.44	30.3	↓
Cardoso et al., 2014	18	14	59.3	12.1	Yes	–	−0.44	5.20	3.44	6.31	7.44	7.41	6.00	9.10	5.89	9.03	23.0	19.2	↑
Schneider and Parente, 2006	40	27	68.0	–	Yes	–	−6.16	–	−2.67	–	−2.44	–	11.6	–	−0.05		–	–	–
Wagner et al., 2009	27	24	69.6	10.4	Yes	–	−0.64	–	−1.38	–	−0.57	–	−1.02	–	0.62		−17.9	17.3	↓
Manes et al., 2011	14	7	65.5	13.9		–	1.56	7.18	0.94	5.85	5.54	6.68	5.36	5.68	8.79	8.52	21.9	19.9	↑
Visagan et al., 2012	35	–	67.3	–	Yes	–	−3.71	7.56	1.31	6.51	1.31	8.98	2.51	9.53	3.60	10.3	5.29	31.35	↑
Buelow et al., 2014	13	7	69.6	15.9	Yes	–	−0.92	6.25	1.85	6.71	2.31	9.38	6.31	8.79	6.77	11.7	15.9	27.92	↑
Damholdt et al., 2011	33	15	68.1	–	Yes	–	−0.61	6.13	1.76	7.79	0.30	7.13	−0.79	8.15	−0.48	9.76	0.18	25.1	↑
Delazer et al., 2009	20	17	71.3	10.3	Yes	–	−4.79	4.57	1.63	5.69	5.72	7.22	7.75	7.14	8.07	8.26	–	–	–
Fein et al., 2007	52	34	73.7	16.2	Yes	Yes	−1.77	11.9	1.09	9.41	3.84	11.8	6.15	11.5	5.98	14.5	15.0	33.4	↑
Bayard et al., 2015	20	11	73.5	11.1	Yes	–	−2.20	3.72	1.10	4.38	2.20	7.70	3.30	8.19	4.50	9.49	8.90	26.1	↑
Wyart et al., 2016	43	17	79.3	20	Yes	–	−2.19	6.13	−0.23	8.27	2.51	8.10	4.56	11.5	4.65	13.1	9.30	30.4	↑
Evans-Roberts and Turnbull, 2010	10	6	71.5	13.6		–	–	–	–	–	–	–	–	–	–	–	29.8	20.9	↑
Bakos et al., 2008	10	9	79.6	14.1	Yes	–	–0.99	–	−3.46	–	–1.86	–	−2.26	–	−6.87	–	−14.6	6.40	↓
Caselli et al., 2011	76	54	75.5	16.1	–	–	−2.63	8.69	3.84	7.88	3.13	9.71	2.34	10.9	2.28	10.6	8.95	29.1	↑
McGovern et al., 2014	36	–	71.6	16.4	Yes	–	−3.58	12.7	2.26	8.81	4.68	9.21	3.46	9.62	0.66	9.45	–	–	–
Perretta et al., 2005	19	8	72.6	14.3	–	–	5.10	–	5.62	–	6.63	–	6.62	–	6.05	–	–	–	–
Alexopoulos et al., 2015	30	0	72.8	16.3	–	Yes	−2.60	9.87	2.60	9.01	5.67	9.61	5.60	9.79	0.87	10.9	12.1	29.4	↑
Pagonabarraga et al., 2007	31	15	70.2	9.90	Yes	–	2.00	–	0.00	–	1.00	–	2.00	–	4.00	–	6.10	17.0	↑
Zamarian et al., 2011	22	11	76.0	9.50	Yes	–	–3.55	6.01	3.91	7.29	4.36	10.6	5.00	9.23	4.55	8.86	14.3	28.8	↑
Sinz et al., 2008	22	17	75.2	10.0	Yes	–	−5.00	4.89	1.64	4.60	6.09	6.03	7.36	7.18	7.00	8.57	17.1	17.5	↑
Delpero et al., 2015	27	14	73.5	9.90	Yes	–	−1.29	2.68	1.00	2.58	4.57	6.48	3.29	6.91	3.07	6.02	–	–	–
Hot et al., 2014	32	19	75.7	–	Yes	–	−2.13	2.00	–0.56	1.26	2.56	2.81	5.19	3.24	5.94	2.88	2.2	5.89	↑
Denburg et al., 2005	40	20	70.7	–	–	Yes	–3.19	6.86	–0.74	7.21	1.03	11.9	−0.86	12.3	1.83	11.2	–	–	–
Huang et al., 2015	65	47	75.3	–	–	Yes	–	–	–	–	–	–	–	–	–	–	–3.48	28.3	↓
Shivapour et al., 2012	116	73	73.6	15.9	Yes	–	–	–	–	–	–	–	–	–	–	–	12.1	38.0	↑
Denburg et al., 2009	79	50	74.0	15.8	Yes	–	–	–	–	–	–	–	–	–	–	–	5.35	–	↑
	1,977	1,081	68.2	13.2	42	4													
Normative data							−1.97	8.59	1.14	10.2	2.90	12.1	3.69	13.2	3.73	11.4	7.55	25.9	

### Task

Seventy-six percent of the studies used the computerized version of the IGT. The remaining studies did not report the procedure associated with task administration (manual vs. computerized), which led to the exclusion of this parameter from the moderator analysis.

None of the included studies rewarded participants based on the IGT performances. Of the five studies reporting payment to participants, only four compensated the participants. Considering the few data points available, compensation was removed from moderator analysis.

### Normative data

The samples from the included studies had an age range of 55–79 years old. Normative data for the total and blocks net scores is presented at Table 1.

### Reversal learning effect

**Figure 2:**
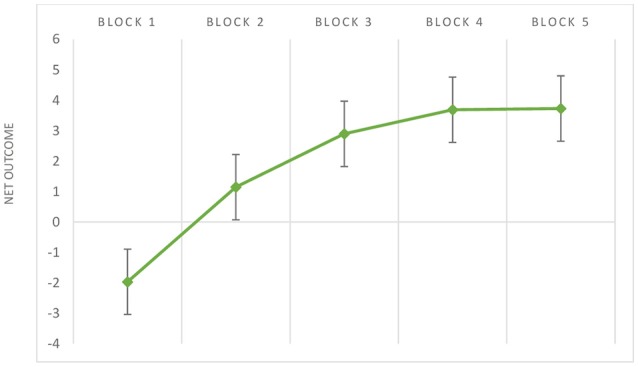
Mean values (and standard errors) of net outcomes (y-axis) considering the performance of older adults across IGT blocks (x-axis).

**Figure 3:**
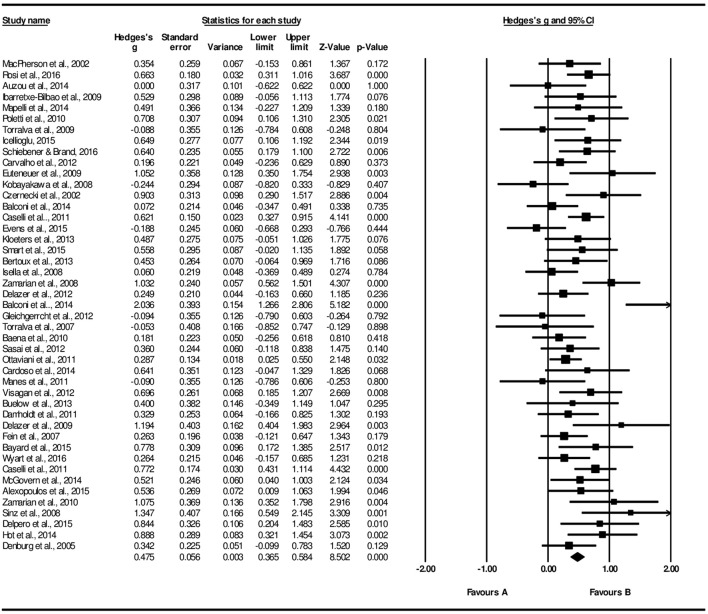
Forest plot of older performance in the initial IGT block (B2-B1).

Figure 2 provides a graphical illustration of the reversal learning effect. There is a significant small-to-medium effect size considering the difference of block 2 performance relative to block 1, *g* = 0.48, 95% CI [0.37, 0.58], *Z* = 8.50, *p* < 0.001. Forest plot of B2-B1 is displayed at Figure 3. The reduced consistency between studies suggests that block 2 still corresponds to an exploratory stage of learning, that is, the trials performed up to this point were not sufficient to learn to discriminate task contingencies.

**Figure 4:**
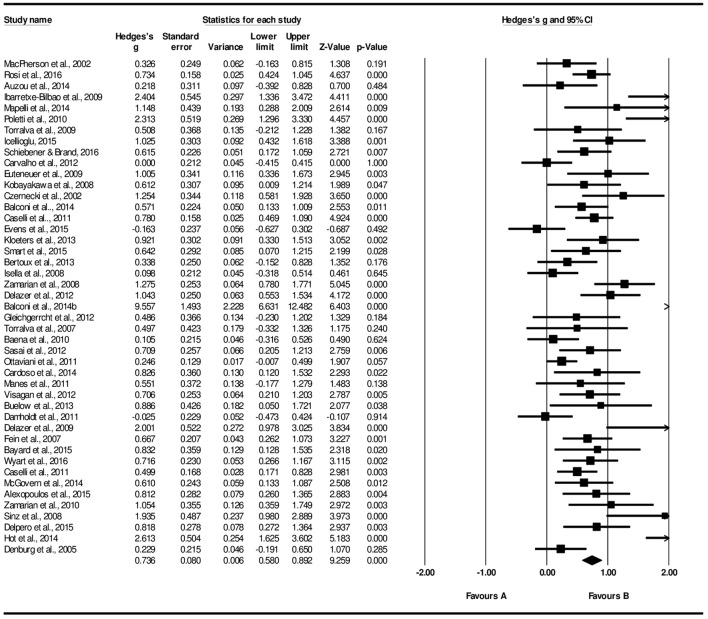
Forest plot of older performance in the final IGT block (B5-B1).

From the block 3 onward, a gradual increase in a medium-to-large effect size is found in relation to block 1: B3-B1, *g* = 0.70, 95% CI [0.56, 0.84], *Z* = 9.70, *p* < 0.001; B4-B1, *g* = 0.73, 95% CI [0.58, 0.89], *Z* = 9.17, *p* < 0.001; B5-B1, *g* = 0.74, 95% CI [0.58, 0.89], *Z* = 9.26, *p* < 0.001. Forest plot of B5-B1 comparison is displayed at Figure 4. The increase in effect size magnitude suggests that, in later blocks, studies systematically report that older adults learn to discriminate advantageous from disadvantageous decks.

The remaining forest plots (B3-B1 and B4-B1) may be found in Figures 5, 6 (Supplementary Information).

### Sensitivity analysis

The sensitivity analysis did not reveal major alterations in the reported effect sizes using either a moderate 0.05 correlation coefficient (B2-B1; *g* = 0.47, 95% CI [0.36, 0.58], *Z* = 8.43, *p* < 0.001; B3-B1, *g* = 0.72, 95% CI [0.60, 0.86], *Z* = 9.91, *p* < 0.001; B4-B1, *g* = 0.77, 95% CI [0.61, 0.93], *Z* = 9.58, *p* < 0.001; B5-B1, *g* = 0.77, 95% CI [0.61, 0.93], *Z* = 9.62, *p* < 0.001), or a 0.08 correlation coefficient (B2-B1, *g* = 0.46, 95% CI [0.35, 0.60], *Z* = 8.36, *p* < 0.001; B3-B1, *g* = 0.71, 95% CI [0.57, 0.85], *Z* = 10.0, *p* < 0.001; B4-B1, *g* = 0.78, 95% CI [0.63, 0.94], *Z* = 9.98, *p* < 0.001; B5-B1, *g* = 0.78, 95% CI [0.63, 0.94], *Z* = 10.0, *p* < 0.001). These results indicate that variation in the actual correlation value would not substantially modify the reported effect sizes and the overall findings of the current meta-analysis.

### Heterogeneity analysis

Significant heterogeneity was found, suggesting variability between studies and demonstrating the importance of accounting for study-level moderators.

B2-B1, *Q*_(44)_ = 89.4, *p* < 0.001, *I*^2^ = 50.8; B3-B1, *Q*_(44)_ = 144.1, *p* < 0.001, *I*^2^ = 69.5; B4-B1, *Q*_(44)_ = 160.1, *p* < 0.001, *I*^2^ = 72.5; B5-B1, *Q*_(44)_ = 89.4, *p* < 0.001, *I*^2^ = 50.8.

### Moderation analysis

None of the moderators described below approached significance, indicating that the heterogeneity between studies is not explained by increased age, differences in years of education or proportion of females.

B2-B1: age, *b* = 0.01, CI 95% [−0.01, 0.03], *Z*_(44)_ = 0.82, *p* = 0.410; years of education, *b* = −0.0002, CI 95% [−0.04, 0.04], *Z*_(35)_ = −0.11, *p* = 0.912; proportion of females, *b* = 0.46, CI 95% [−0.27, 1.19], *Z*_(35)_ = 1.22, *p* = 0.221.

B3-B1: age, *b* = −0.004, CI 95% [−0.03, 0.02], *Z*_(44)_ = −0.28, *p* = 0.777; years of education, *b* = 0.0038, CI 95% [−0.05, 0.05], *Z*_(35)_ = 0.15, *p* = 0.880; proportion of females, *b* = 0.37, CI 95% [–0.60, 0.34], *Z*_(35)_ = 0.75, *p* = 0.453.

B4-B1: age, *b* = −0.004, CI 95% [−0.04, 0.03], *Z*_(44)_ = −0.26, *p* = 0.795; years of education, *b* = −0.004, CI 95% [−0.06, 0.049], *Z*_(35)_ = −0.15, *p* = 0.882; proportion of females, *b* = 0.0007, CI 95% [−1.07, 1.07], *Z*_(35)_ = 0.00, *p* = 0.999.

B5-B1: age, *b* = −0.005, CI 95% [−0.04, 0.03], *Z*_(44)_ = −0.29, *p* = 0.770; years of education, *b* = −0.004, CI 95% [−0.06, 0.05], *Z*_(35)_ = −0.16, *p* = 0.873; proportion of females, *b* = 0.0018, CI 95% [−1.06, 1.06], *Z*_(35)_ = 0.00, *p* = 0.997.

## Discussion

The IGT is one of the most widely used tools to assess decision-making. However, most of the research on IGT and aging has been mainly focused on the performance comparison between older adults and clinical or younger groups. Despite the evidence that older adults make more disadvantageous decisions than younger groups on the IGT (Mata et al., 2011), one question remains unclear: do older adults learn to choose advantageously along the task?

The trend to collapse the choices across blocks to create a summary score—the total net score—restricts the understanding of learning processes that may take place during the IGT. In fact, and to our knowledge, this study is the first to consider within-subject methods when meta-analyzing the IGT performance in different blocks. This method is a relevant contribution to the research field of decision-making in older-adults, as performance in IGT may be ruled by two distinct types of decision-making—under uncertainty and under risk—and only the first is found to be impaired in older adults (Mata et al., 2011). Therefore, it is critical to understand if older adults' decisions remain ruled by uncertainty or, in turn, older adults are capable to learn from experience and move to decisions based on known outcomes.

During the first blocks, the decision-making on the IGT is expected to be driven by affective cues. This is an exploratory stage of learning, as participants have not yet deciphered the contingencies of the decks, and decision-making is made under uncertainty (Brand et al., 2007). Confirming the exploratory process of learning, a negative block net score on block 1 stands out in the older group. Right after block 1, a significant reversal learning effect was found. However, the effect size in relation to the difference between block 2 and block 1 was only small-to-medium in magnitude (*g* = 0.48), demonstrating that a significant improvement in performances is not a robust finding across studies.

From trial 50 onward, choices are expected to be more adaptive and driven by the acquired knowledge (Bechara, 2007). The decision is now expected to be made under risk, as the contingencies of the task are expected to be learned (Brand et al., 2007). The effect sizes from block 3 to block 5 became medium-to-large in magnitude (*g* = 0.70 to 0.78), which is in line with the literature defining the trial 50 as the starting point to develop adaptive choices on the IGT (Bechara, 2007).

The main findings provide evidence that older adults exhibit an advantageous pattern of performance during the IGT. The robust reversal learning effect evidenced in block 3 suggests that the shift from ambiguity to risk seems to occur in this block, and importantly, around the trial proposed by Bechara and colleagues (Bechara, 2007). The hypothesis that older adults, compared to younger groups, tend to choose immediately attractive options on IGT that lead to higher monetary losses along the task (Mata et al., 2011) does not necessarily mean that older adults are not capable to learn under uncertainty. The within-subjects design of our meta-analysis highlights that learning processes under uncertainty are not entirely compromised with increased age. Moreover, our analyses illustrate how collapsing the choices in a total net score might hide the reversal learning effect across the blocks, masking the older adults' ability to learn under uncertainty.

From our results, older adults seem to be able to use the salient affective stimuli and then integrate these somatic markers (Damasio, 1994) in memory and rational analytical systems, albeit in a less effective way than younger adults (Frank and Kong, 2008; Hämmerer et al., 2011; Mata et al., 2011).

The reviewed studies indicate that older adults show a positive net outcome while performing the task, which means that they finish the task with an adaptive pattern of decision-making, by choosing the advantageous decks more frequently. Only 8 of the 43 studies reported a negative performance in older adults. Despite the positive outcome evidenced by older adults, it should be acknowledged that a net score of ≥10 is the cut-off index that describe performances that are not within the range of vmPFC patients (Bechara and Damasio, 2002; Bechara et al., 2002). The total net normative value of older adults is below 10 by Bechara's criterion (Bechara et al., 2002), which would suggest impaired performance and a “myopia for the future” in older groups. The variance around the mean must be taken, however, into account (±25.9), as well as individual differences.

Direct evidence from the 8 studies reporting negative net scores (Bakos et al., 2008; Wagner et al., 2009; Ottaviani and Vandone, 2011; Bertoux et al., 2013; Auzou et al., 2014; Evens et al., 2015; Schiebener and Brand, 2016) may help to identify relevant individual differences implicated in IGT performance, since the moderators systematically reported in the literature failed to achieve significance in our meta-analysis. This comprehensive analysis is limited, however, by the focus of the included studies on group differences. The focus of the majority of the studies reporting negative net outcomes is redirected to variables that explain impaired performance in clinical groups as opposed to a comprehensive interpretation of the performance of healthy older groups. Nevertheless, Bakos et al. (2008) observed that the oldest old group exhibited poor decision-making in IGT compared to the younger elderly group, despite similar performance in selective attention, short-term memory, and working memory. This finding would suggest that increasing age may compromise adaptive performance in IGT, but our meta-regression showed that age did not moderate the findings. In turn, Schiebener and Brand (2016) included an age range of 18–86 years. Remarkably, age-related variance on IGT performance occurred only in the last 60 trials and in a task with explicit instructions, that is, when decisions are expected to be conducted under risk (Schiebener and Brand, 2016). In the first 40 trials—decision-making under uncertainty—the association between increasing age and less advantageous decision-making was small. This main finding is in line with the theoretical background of the current meta-analysis, highlighting that economic decision-making in later life shows specific dynamics.

Under uncertainty, the amygdala is a critical brain area to trigger affective cues and respond to primary inducers (Damasio, 1994; Hsu et al., 2005) and, interestingly, age does not seem to significantly affect this structure (Mather et al., 2004). This may explain why older adults are capable of deciding advantageously under uncertainty. The difficulty in achieving a performance similar to the younger group, as previously documented in Mata et al.'s (2011) meta-analysis, may be explained by a functional deficit in cognitive functioning. Schiebener and Brand (2016) reported that, after controlling for the effects of cognitive abilities, no age-related variance in decision-making in the IGT remained. This result suggests that age-related changes in EF and reasoning may explain individual differences in IGT performance.

The differences between older and younger adults may be further explained by the difficulty in the older group to persecute the option more likely to be rewarded when differences in reward likelihood are small (Hämmerer et al., 2011). Steingroever et al. (2013) argued that 3 of 4 decks seem to present too similar outcomes. Reduced and similar FRN amplitude in the processing of gains and losses in older adults was previously documented (Hämmerer et al., 2011).

Age-related effects on risky decision-making extends beyond cognition and is also linked to individual differences in personality. Denburg et al. (2009) found that high levels of trait neuroticism in older adults (i.e., proneness to experience negative affective states such as fear, anxiety, sadness, guilt, and anger) is associated with impaired decision-making performance. Importantly, younger adults with high trait-anxiety (Suhr and Tsanadis, 2007) and negative affect (Miu et al., 2008) also show impaired decision-making under uncertainty, despite the increased and potentially adaptive anticipatory somatic signals associated with high trait-anxiety (Suhr and Tsanadis, 2007). The main findings suggest that affect and personality are critical mechanisms to extend our knowledge on older adults' performance in the IGT and, therefore, studies should include these variables.

From the empirical evidence accumulated along the years, the normative total net score for older groups is of 7.55 (±25.9). The lack of age moderation effects suggests that the proposed normative score is representative of the 55–79 age range. The calculation of normative scores, even limited to a statistical criterion, constitute a group reference to compare individual performances of IGT in healthy older adults. Future studies may cluster impaired and unimpaired performances from *Z* scores. The Z scores represent the number of standard deviations below or above the mean considering the individual total net score. Moreover, performance may be analyzed depending on whether the overall score is significantly different from the normative pooled mean, in a negative or positive direction, using the binomial test (Siegel, 1956; Damasio, 1994; Denburg et al., 2005). Under the assumption that a total net score of zero reflects equal probability to choose advantageous and disadvantageous decks, impaired performance is significantly different from zero in a negative direction, while unimpaired performance differs significantly in a positive direction (Denburg et al., 2005). The participants whose total score is not statistically significant from zero in either direction may be included in the borderline group (Denburg et al., 2005).

In sum, our results contradict the assumption (Kovalchik and Allman, 2006) that older adults engage in a random selection strategy, since older adults tend to evidence a pattern of advantageous decision-making. Furthermore, our meta-analysis point that the performance within-study variability reported by Steingroever et al. (2013) contrasts with a robust effect size between-studies. Steingroever et al. (2013) proposed that 100 trials were not sufficient to learn to discriminate safe from risky options and, subsequently, the switch behavior from exploration to exploitation would not occur. From our data, older adults seem to first explore the different decks, as evidenced in negative net outcomes in block 1, and then exploit the most profitable options, culminating in positive net outcomes from block 2 onward. The reversal learning effect is consistently found around block 3. These results are line with Bechara's (Bechara et al., 1994; Bechara, 2007) assumption that after 50 selections participants tend to choose the long-term attractive decks. In the later blocks of IGT, older adults decide in some extent toward less risky choices, suggesting that older adults do not remain unconditionally under uncertainty. In turn, the selection strategies seem to be guided in some way by explicit rules acquired in the course of the task.

From our findings, we propose that decision-making on IGT and aging moves toward uncertainty—where the outcomes are unknown—to risk—where the outcomes were learned and may be used to guide adaptive economic decisions. Differences between younger and older groups found in previous studies may be explained by in a great extent by age-related changes in brain areas associated with cold EF and not, necessarily, with impaired reversal learning.

### Limitations

This meta-analysis has some limitations that must be taken into account when interpreting the results. Despite the efforts to include gray literature, publication bias was found in the current systematic search, indicating a possible overestimation of the results.

Correlation coefficients between blocks were further imputed since the values means and standard deviations of block net scores were not reported on the original statistical analysis. Importantly, the sensitivity analysis did not alter the overall findings of the meta-analysis. We strongly recommend authors using the IGT in their research to report correlation values between blocks, as well as all the variables systematically identified in the literature thought to modulate IGT performance.

In the current meta-analysis, the included studies were too heterogeneous, but the moderators with satisfactory data points were non-significant. Since the moderators explaining the heterogeneity remain unknown, the use of the normative data and the generalization of the findings may be compromised. A detailed description of variables relevant to assess IGT performance would allow to explore systematically not only the variables accounting for the heterogeneity between-studies, but also to explain the idiosyncrasies on performances evidenced by Steingroever et al. (2013).

### Future directions

Steingroever et al. (2013) proposed that the frequency of losses is an important variable to explain performance on the IGT, given that participants seem to prefer the decks with infrequent losses. Our meta-analysis did not allow to test the trial-to-trial behavior adjustment after losses and gains, again due to the lack of available information. The analysis of gains and losses ratio in function of decks selection is of high relevance to increased caution after losses (Rolison et al., 2013, 2016). This would also help to clarify the pattern of strategies of older adults that may be associated with reduced total net scores.

The existing meta-analyses should also be extended to explore the reversal learning effect in younger (children, adolescents, and younger adults) and clinical populations. Although older adults show a robust learning effect on the expected block, in light of a previous meta-analysis (Mata et al., 2011) it would be important to explore if the elderly need more trials to overcome the initial uncertainty than younger adults. Since a reversal learning effect is observed, we would expect that once under risk (i.e., when the task contingencies were learned) an equivalent performance would be achieved. However, the reversal learning effect may be faster in younger groups, giving them an advantage to reach a more positive total net outcome on the IGT.

Research focused on personality and the IGT also has to be extended to older groups, as it is likely that individual differences modulate age-related changes in IGT performance.

## Author contributions

Conceptualization and paper review: RP, AG, CF, FB, FF, and JM. Data codification: RP and AG. Data analysis: RP and FF. Paper preparation: RP. Supervision: CF, FB, FF, and JM.

### Conflict of interest statement

The authors declare that the research was conducted in the absence of any commercial or financial relationships that could be construed as a potential conflict of interest.

## Footnotes

^1^Despite systematic search procedures, meta-analyses often evidence publication bias since studies reporting significant differences and large effects, are more likely to be published than studies that report non-significant differences (e.g., Dickersin, 2015). To assess the overestimation bias in the reported effect sizes we calculated the Egger's (1997) regression intercept for each block comparison. Egger's intercept was not significant in B2-B1 (*B* = 1.04, *p* = 0.172). However, a significant intercept was found in B3-B1 (*B* = 2.97, *p* < 0.001), B4-B1 (*B* = 3.59, *p* < 0.001), and B5-B1 (*B* = 3.58, *p* < 0.001), suggesting the existence of publication bias.
