# Supplementary figures and images for: Meta-Analytic Evidence for a Reversal Learning Effect on the Iowa Gambling Task in Older Adults
Source: Front Psychol. 2017 Oct 11;8:1785. doi: 10.3389/fpsyg.2017.01785 (PMC5641897; doi:10.3389/fpsyg.2017.01785)

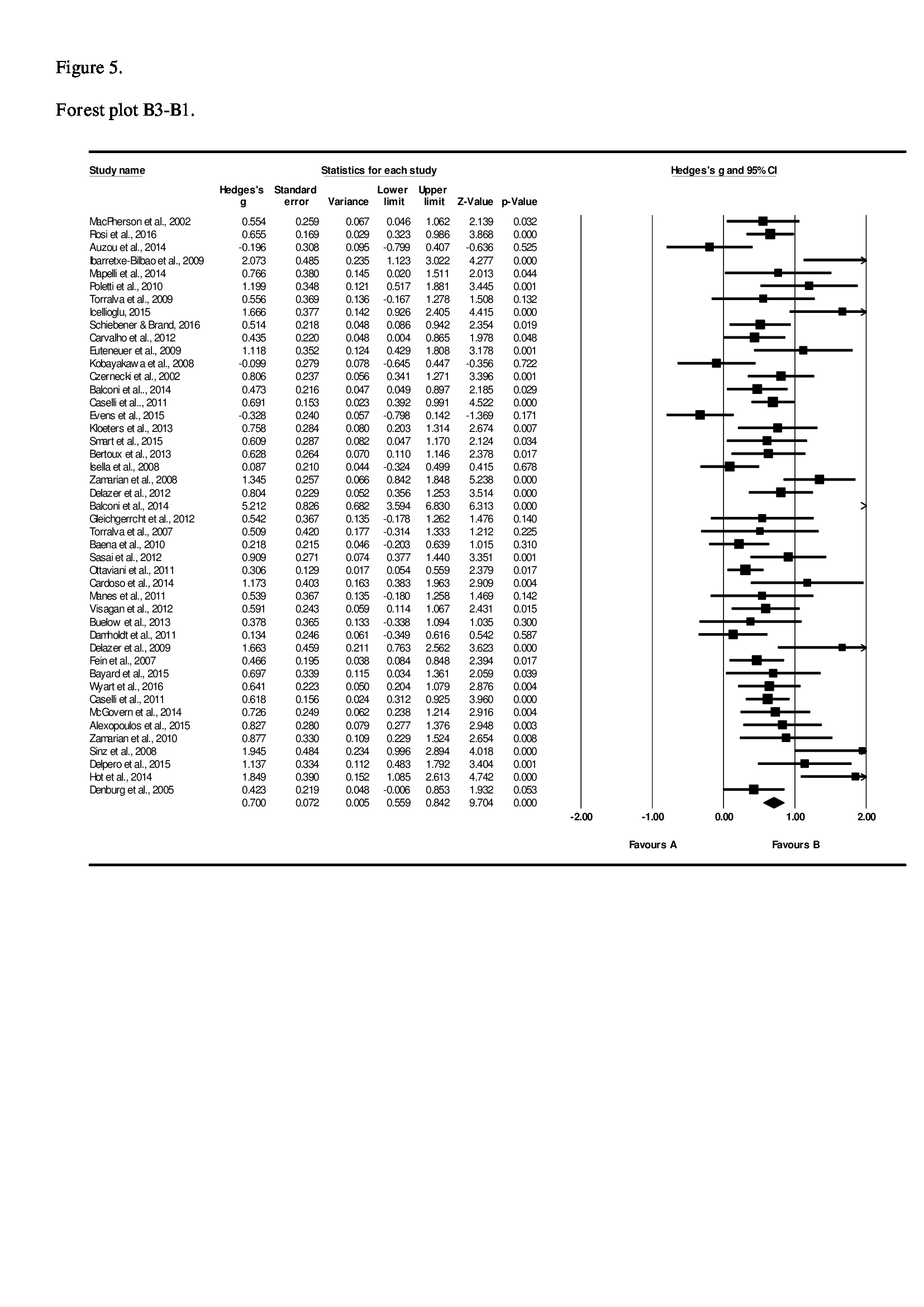

Supplement: Supplementary file 1 [file Image1.JPEG]

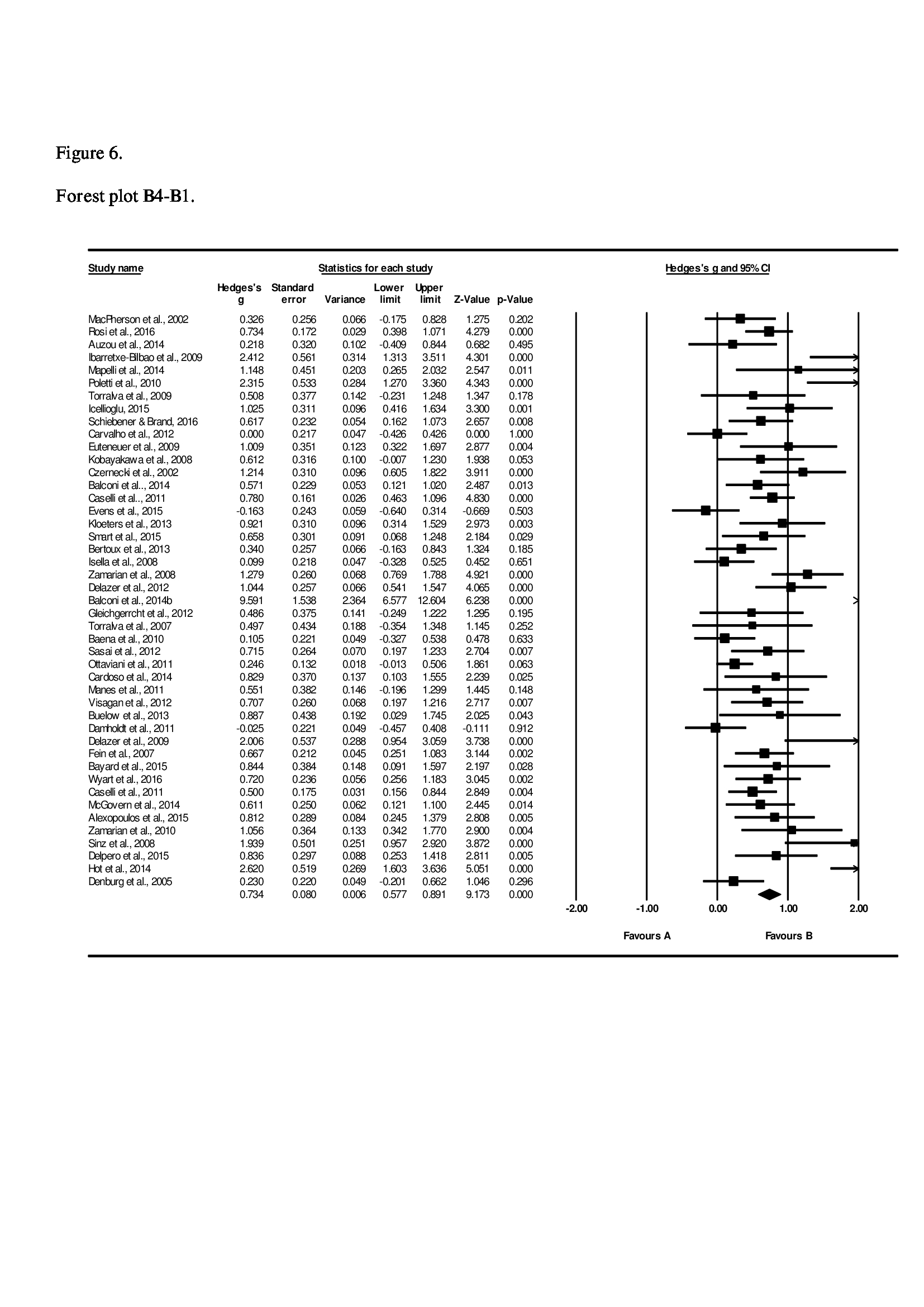

Supplement: Supplementary file 2 [file Image2.JPEG]
